# Supplementary material for: Splice-Junction-Based Mapping of Alternative Isoforms in the Human Proteome
Source: Cell Rep. Author manuscript; Available in PMC 2020 Jan 15. (PMC6961840; doi:10.1016/j.celrep.2019.11.026)
Supplement: 2 [file NIHMS1546469-supplement-2.zip › DF1/PXD006675/Aorta/Aorta_12_REX1BD_FDAESAGPAAVDGDAR.pdf]

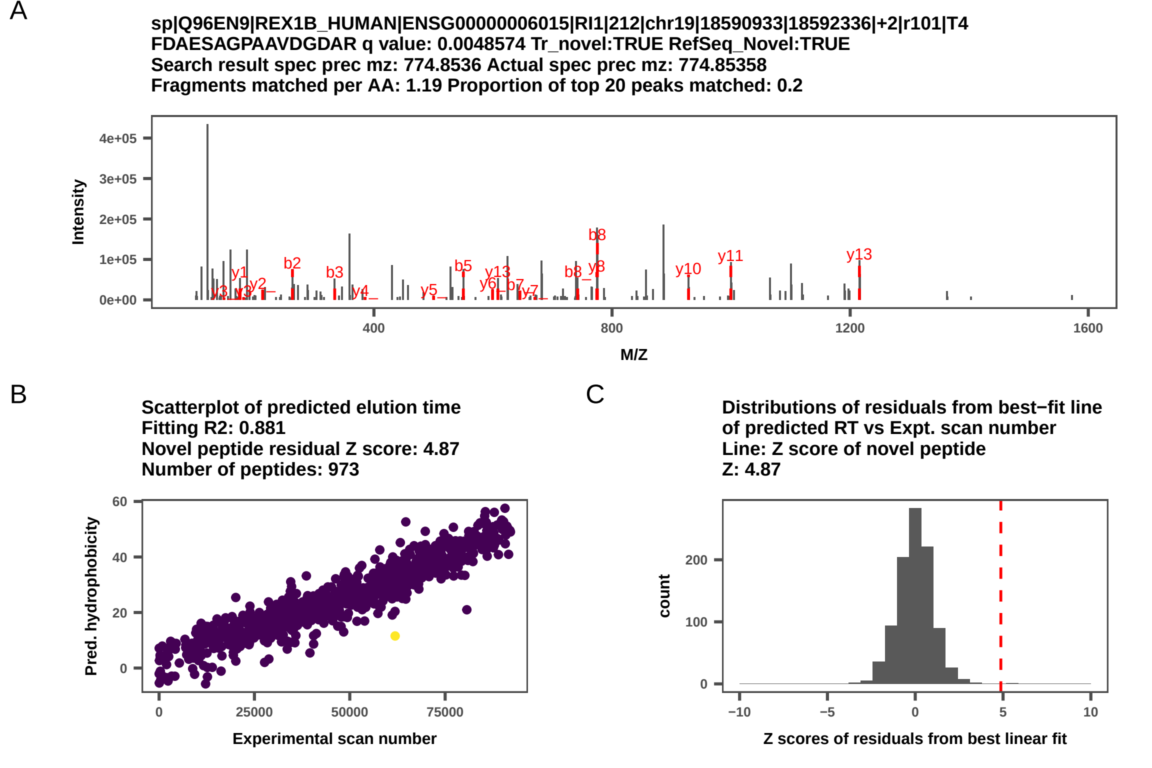
A
sp|Q96EN9|REX1B_HUMAN|ENSG00000006015|RI1|212|chr19|18590933|18592336|+2|r101|T4
FDAESAGPAAVDGDAR q value: 0.0048574 Tr_novel:TRUE RefSeq_Novel:TRUE
Search result spec prec mz: 774.8536 Actual spec prec mz: 774.85358
Fragments matched per AA: 1.19 Proportion of top 20 peaks matched: 0.2
Intensity
4e+05
3e+05
2e+05
1e+05
0e+00
b8
y13
y11
b2
b5
y10
b3
y1
y13
b8
y8
y2
y6
b7
y3
y3
y4
y5
y7
400
800
1200
1600
M/Z
B
Scatterplot of predicted elution time
Fitting R2: 0.881
Novel peptide residual Z score: 4.87
Number of peptides: 973
Pred. hydrophobicity
60
40
20
0
0
25000
50000
75000
Experimental scan number
C
Distributions of residuals from best−fit line
of predicted RT vs Expt. scan number
Line: Z score of novel peptide
Z: 4.87
count
200
100
0
−10
−5
0
5
10
Z scores of residuals from best linear fit
